# Phylogeography and invasion history of *Aedes aegypti*, the Dengue and Zika mosquito vector in Cape Verde islands (West Africa)

**DOI:** 10.1111/eva.12834

**Published:** 2019-08-03

**Authors:** Patrícia Salgueiro, Célia Serrano, Bruno Gomes, Joana Alves, Carla A. Sousa, Ana Abecasis, João Pinto

**Affiliations:** ^1^ Global Health and Tropical Medicine (GHTM), Instituto de Higiene e Medicina Tropical (IHMT) Universidade Nova de Lisboa (UNL) Lisboa Portugal; ^2^ Oswaldo Cruz Institute (IOC) Fundação Oswaldo Cruz (FIOCRUZ) Rio de Janeiro Brasil; ^3^ Direção Geral de Saúde/Instituto Nacional de Saúde Pública, Ministério da Saúde de Cabo Verde Praia Cabo Verde

**Keywords:** *Aedes aegypti*, Africa, Cape Verde, Dengue, phylogeography, population genetics, vector control, Zika

## Abstract

*Aedes*‐borne arboviruses have spread globally with outbreaks of vast impact on human populations and health systems. The West African archipelago of Cape Verde had its first outbreak of Dengue in 2009, at the time the largest recorded in Africa, and was one of the few African countries affected by the Zika virus epidemic. *Aedes aegypti* was the mosquito vector involved in both outbreaks. We performed a phylogeographic and population genetics study of *A. aegypti* in Cape Verde in order to infer the geographic origin and evolutionary history of this mosquito. These results are discussed with respect to the implications for vector control and prevention of future outbreaks. Mosquitoes captured before and after the Dengue outbreak on the islands of Santiago, Brava, and Fogo were analyzed with two mitochondrial genes COI and ND4, 14 microsatellite loci and five *kdr* mutations. Genetic variability was comparable to other African populations. Our results suggest that *A. aegypti* invaded Cape Verde at the beginning of the Holocene from West Africa. Given the historic importance of Cape Verde in the transatlantic trade of the 16th–17th centuries, a possible contribution to the genetic pool of the founding populations in the New World cannot be fully discarded. However, contemporary gene flow with the Americas is likely to be infrequent. No *kdr* mutations associated with pyrethroid resistance were detected. The implications for vector control and prevention of future outbreaks are discussed.

## 1 INTRODUCTION

*Aedes*‐borne arboviral diseases have become a major global health concern. Dengue is the most important mosquitoborne viral disease in the world, causing 10,000–20,000 deaths per year (WHO, 2012) and almost 400 million new infections are estimated to occur annually (Bhatt et al., 2013). In 2015, a new outbreak caused by Zika virus has affected 84 countries (WHO, 2017) with more than 200,000 confirmed cases just in the Americas (Mitchell, 2016). Zika virus has been associated with severe congenital neurological abnormalities in infants born to mothers infected during pregnancy, reported in 31 countries (WHO, 2017). For these arboviruses, a proportion of cases is asymptomatic (Bhatt et al., 2013; Petersen et al., 2016), which together with poor disease surveillance and lack of point‐of‐care diagnostic tests may be potentially misdiagnosed and underreported (Wilder‐Smith & Byass, 2016). This is particularly important in Africa where the majority of febrile illnesses are treated presumptively as malaria (Amarasinghe, Kuritsky, Letson, & Margolis, 2011; Stoler & Awandare, 2016). As a consequence, little is known about the epidemiology of these arboviruses in the African continent (Amarasinghe et al., 2011; CDC, 2015; Nutt & Adams, 2017; Sang & Dunster, 2001; Were, 2012). The control of Dengue and Zika is largely dependent on efficient and sustainable vector control measures.

Arboviruses like Dengue, Zika, and Yellow Fever have spread worldwide following the expansion of their main vector *Aedes aegypti*. This mosquito has originated in Africa from an ancestral sylvatic and more zoophilic form *A. aegypti formosus*, which expanded from tropical forests to urban areas giving rise to a domestic and anthropophilic form known as *A. aegypti aegypti* (Bennett et al., 2016; Brown et al., 2011; Moore et al., 2013; Powell & Tabachnick, 2013). This form was the only that succeeded in invading the rest of the world, forming a monophyletic group (Bennett et al., 2016; Brown et al., 2014, 2011; Gloria‐Soria et al., 2016). *Aedes aegypti* arrived to the New World together with the first Europeans and Africans during the historical transatlantic shipping traffic between 1500s and 1700s, followed by the first reports of Yellow Fever and Dengue in the region (Powell & Tabachnick, 2013).

Genetic evidence has confirmed a single out of Africa colonization event for *A. aegypti* and its historic route of expansion throughout the world (Bennett et al., 2016; Brown et al., 2014, 2011; Moore et al., 2013).

After the invasion of the Americas, a cline of reduced genetic variability suggests spreading westwards with subsequent founder events, from the American continent to Asia and Oceania (Brown et al., 2014; Gloria‐Soria et al., 2016). A mitochondrial DNA study reported two ancestral clades from which *A. aegypti* populations outside Africa have arisen (Moore et al., 2013), one associated with West Africa and another with East Africa. Nevertheless, a scenario where populations outside Africa originated from a single sample with the two mtDNA lineages from ancestral Africa cannot be discarded (Gloria‐Soria et al., 2016). Presently in Africa, both *formosus* and *aegypti* subspecies co‐occur and interbreed. The only exception seems to be a rural/forest population from the Rabai District in Kenya, where the two subspecies remain genetically distinct (Brown et al., 2014, 2011; Gloria‐Soria et al., 2016). Most likely due to increasing urbanization, populations of *A. aegypti* in Africa can now be found in many urban settings (Kamgang et al., 2013; Paupy et al., 2008) even if they fall genetically into the *formosus* group (Gloria‐Soria et al., 2016). A study (Crawford et al., 2017) using exome sequences proposed West Africa, in particular Senegal, as the source of the America invasion by *A. aegypti*. Another recent study (Kotsakiozi et al., 2018), based on SNPs from 20 African populations, suggested Angola as the most likely origin.

**Figure 1:**
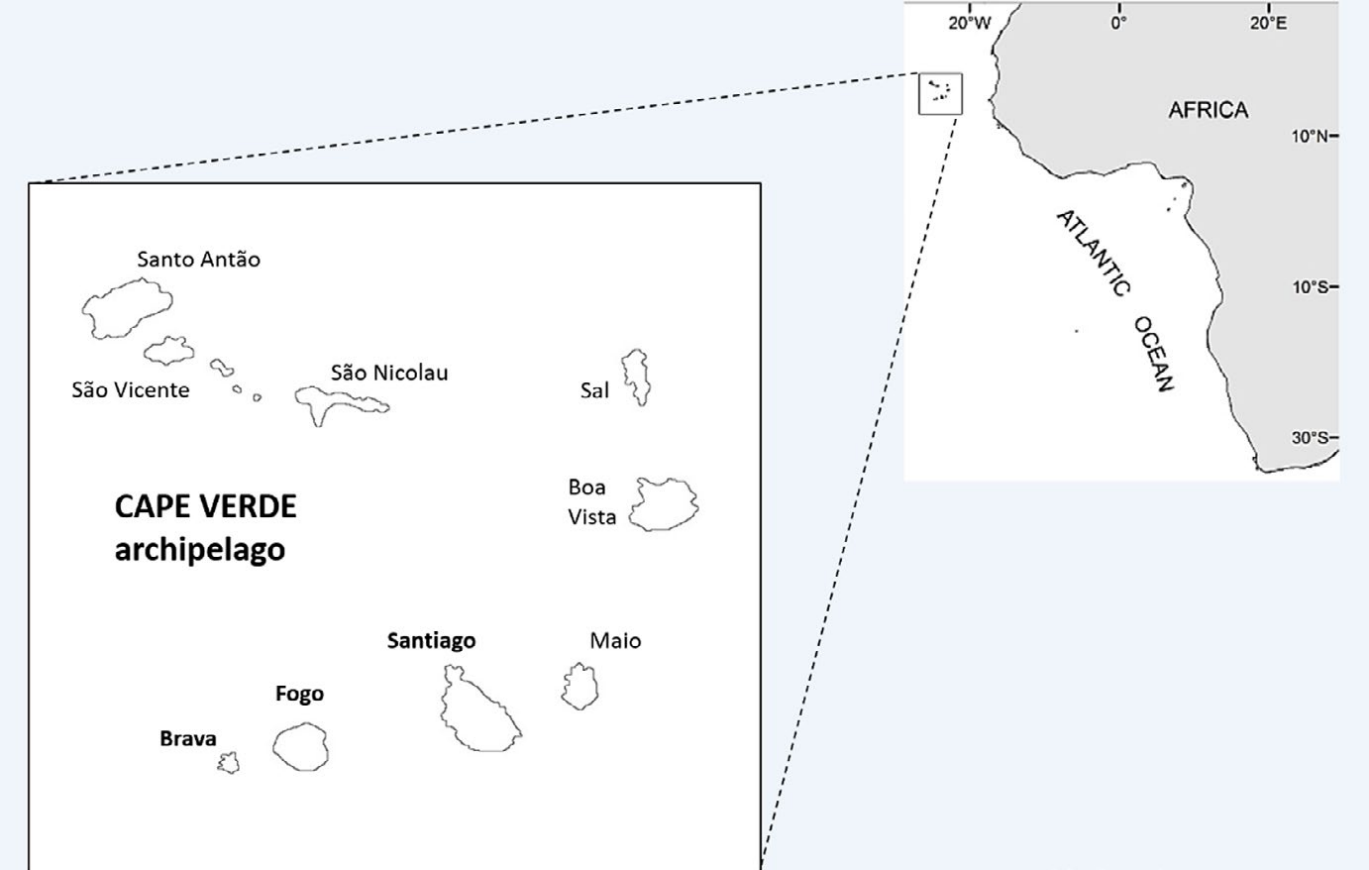
Map with the location of the Cape Verde archipelago, naming the nine inhabited islands. The *Aedes aegypti* samples from the present study have been collected from the islands in bold (Santiago, Brava, and Fogo)

The archipelago of Cape Verde is located at around 500 km West of Senegal (West Africa). It comprises nine inhabited islands (Figure 1) and a current local population of about 500,000 inhabitants. Cape Verde islands had no human occupation when they were first reached by the Portuguese in the 1450s (Lobban, 2018). During most of the 16th and 17th centuries, the islands served as port‐of‐call in a transatlantic commercial slave network (Russell‐Wood, 1998), where most of the ships from West Africa to Santiago island would continue to the New World (Lobban, 2018; Russell‐Wood, 1998).

Dengue was reported for the first time in Cape Verde at the end of September 2009, in an outbreak considered at the time the largest ever recorded in Africa, having affected all islands. Over 20,000 cases (about 5% of the country population), 174 hemorrhagic fever cases and four deaths were reported (WHO, 2009). Dengue type‐3 virus (DENV‐3) was confirmed both in Cape Verde and in the concomitant dengue epidemic in Senegal (Franco et al., 2010). In 2015, DENV‐2 and DENV‐4 were found circulating in field mosquito samples collected in Santiago Island (Guedes et al., 2017). In October 2015, an unprecedented epidemic of Zika virus was reported in Cape Verde (WHO, 2015), with more than 7,500 suspected cases. By August 2016, 18 microcephaly cases were reported in the islands of Fogo, Santiago, and Maio (Monteiro, 2016). This was the first time that a Zika strain associated with neurological damage in infants was detected in Africa (WHO, 2016). Given this fact, the timing of the epidemic and the high number of travelers visiting Cape Verde from the Americas, it was suggested that the outbreak was likely caused by the Asian genotype circulating there (Lourenço et al.., 2018).

*Aedes aegypti* is the only mosquito vector of these arboviruses so far detected in Cape Verde. This species was reported for the first time in S. Vicente Island and later on in all islands since 1964 (Ribeiro, Ramos, Capela, & Pires, 1980). Morphological identification of the subspecies was attempted with specimens from a single island (Santiago), and only *A. a. formosus* was detected (Vazeille et al., 2013). Low vector competence has been associated with African strains of *A. aegypti*, particularly *A. a formosus*, with a lower susceptibility to DENV‐2 (Diallo et al., 2013; Sylla, Bosio, Urdaneta‐Marquez, Ndiaye, & Black, 2009). However, *A. aegypti* from Santiago Island collected in 2010 showed a moderate ability to transmit the epidemic DENV‐3, and high susceptibility to chikungunya and yellow fever viruses (Vazeille et al., 2013). A more recent study based on collections carried out in 2012 found that mosquitoes displayed higher vector competence for DENV‐2 and DENV‐3 when compared to DENV‐1 and DENV‐4 (Moura et al., 2015).

In Cape Verde, integrated vector control strategies have been directed to both malaria and dengue vectors, *Anopheles arabiensis* and *A. aegypti*, respectively, through source reduction, diesel use, biological control with fish (*Gambusia sp*.), and chemical control with the insecticides temephos and deltamethrin (Ministry of Health Cape Verde, WHO, & University of California, 2012; de Pina, 2013). The efficacy of insecticide‐based vector control has been threatened by the evolution of insecticide resistance worldwide (Smith, Kasai, & Scott, 2016). Insecticide susceptibility tests performed in *A. aegypti* from Santiago island during the dengue outbreak in 2009 revealed resistance to DDT but susceptibility to pyrethroids (Dia et al., 2012). Later on, *A. aegypti* mosquitoes collected in the same island in 2012 already exhibited resistance to pyrethroids (deltamethrin, cypermethrin) and to the organophosphate temephos (Rocha et al., 2015). A major resistance mechanism affects a gene in the insect's voltage‐gated sodium channel. In this gene, several mutations, known as knockdown resistance (*kdr*) mutations, have been described and associated with DDT and pyrethroids resistance worldwide (Du, Nomura, Zhorov, & Dong, 2016). These mutations were not detected in *A. aegypti* collected in 2012 (Rocha et al., 2015).

Since Cape Verde is in a strategic route linking Africa, Europe, and the Americas, by sea or by air, thus having a high risk of introduction of new strains of arboviruses or new vectors, it is of great importance to study the local population structure of *A. aegypti*. Therefore, we have developed a comprehensive phylogenetic and population genetics study of *A. aegypti* from three islands of Cape Verde with samples collected before and after the dengue outbreak of 2009. We have measured levels of genetic variability and determine the population structure, inferred the evolutionary history and detect possible origins for this insular population, and evaluated the allelic composition in three *kdr* sites.

## 2 MATERIALS AND METHODS

### 2.1 Mosquito sampling

*Aedes aegypti* mosquitoes were collected between November and December 2007 in three islands of the leeward group of the Cape Verde archipelago: Santiago, Fogo, and Brava and in April 2010 from Santiago island (Figure 1, Table 1). The samples consisted of larvae or pupae collected from 12 breeding sites. These were reared to adult stage and then morphologically identified to species (Ribeiro & Ramos, 1995; Ribeiro et al., 1980). The detailed procedure of the entomological survey has been described elsewhere (Alves et al., 2010). Adult specimens were individually preserved in tubes filled with silica gel and kept at room temperature until DNA extraction. Genomic DNA was extracted using a phenol: chloroform protocol (Donnelly, Cuamba, Charlwood, Collins, & Townson, 1999).

**Table 1:** Number of *Aedes aegypti* mosquitoes used for different analysis per year and collection site

Year	date	Island	Site	DNA extracted	COI	ND4	*kdr*	Microsatellites
2007	November–December	Santiago	Praia	53	22	21	47	47
		Brava	Nª Srª do Monte	6	6	6	–	–
		Fogo	S. Filipe	1	1	1	–	–
2010	April	Santiago	Barragem do Poilão	27	21	14	27	27
Total				87	50	42	74	74

### 2.2 Mitochondrial DNA sequencing and phylogenetic analysis

We have analyzed two mitochondrial genes: cytochrome oxidase subunit I gene (COI) and NADH dehydrogenase subunit 4 gene (ND4). The gene COI was amplified with primers published by (Paupy et al., 2012). The gene ND4 was amplified using the primers described by Paduan and Ribolla (2008). PCR conditions for both genes were optimized by Seixas et al. (2014).

PCR products were purified and sequenced directly with the PCR primers (forward and reverse sequences obtained for each individual). Only sequences with no double peaks, suggestive of nuclear mtDNA segments (or NUMTs), were subsequently analyzed. Sequences were aligned and numbered in reference to the *A. aegypti* complete COI sequence (bases 1–1537, GenBank accession number AF390098 (Morlais & Severson, 2002)) and ND4 (bases 1–1344, GenBank accession number DQ440274 (da Costa‐Ribeiro, Lourenço‐De‐Oliveira, & Failloux, 2007)).

Summary statistics, including the number of haplotypes (Hap), haplotype diversity (Hd), nucleotide diversity (*π*), standard neutrality tests: Tajima's D (Tajima, 1989), Fu's Fs (Fu, 1997), and mismatch distribution (Schneider & Excoffier, 1999), were calculated using ARLEQUIN v.3.5 (Excoffier & Lischer, 2010). When the neutrality test values were negative and significant and the mismatch distribution curve was unimodal, the population was considered to fit the sudden expansion model. Then, the expansion time was estimated (Rogers & Harpending, 1992) assuming a substitution rate of 0.023 (COI) and 0.0125 (ND4) (Brower, 1994; Yu et al., 1999) and generation time of 15 generations per year (Crawford et al., 2017). DNAsp 6 (Rozas et al., 2017) was used to identify unique haplotypes.

The haplotypes obtained from the mosquito population of Cape Verde were analyzed together with other sequences of the same genes available in GenBank, including sequences of *Aedes albopictus* used as outgroup. The 368 ND4 and 57 COI sequences are detailed in Appendixes 1 and 3, respectively. In order to increase the representativeness of African samples, other COI sequences (Bennett et al., 2016) were included in the study, resulting in a new alignment of 94 COI sequences but with only 493 base pairs (bp) (Appendix S2).

We used ModelFinder (Kalyaanamoorthy, Minh, Wong, Haeseler, & Jermiin, 2017) implemented in IQ‐TREE web server (Trifinopoulos, Nguyen, von Haeseler, & Minh, 2016) to determine the best fitting substitution model.

Bayesian Markov chain Monte Carlo inference, as implemented in BEAST 1.8.4 (Drummond, Suchard, Xie, & Rambaut, 2012), was used to reconstruct the phylogenies that best describe the evolutionary history of the data set, given the sequences and a priori information of the geographic traits. The substitution model HKY (Hasegawa, Kishino, & Yano, 1985) with gamma and invariant sites and three partitions into codon positions were used. Furthermore, a Bayesian skyline population growth model was used (Drummond, Rambaut, Shapiro, & Pybus, 2005) which allows for flexibility in demographic reconstruction and a strict molecular clock was assumed with a substitution rate of 0.023 for COI and 0.0125 for ND4, as previously described (Brower, 1994; Yu et al., 1999). The analyses were run in 3–5 separate independent runs in BEAST with 100,000,000 generations, sampled every 50,000 runs (ND4), 100,000,000 sampled every 10,000 runs (COI). To analyze convergence and stability, we used TRACER v1.6 software (Rambaut, Drummond, Xie, Abebe, & Suchard, 2017).

TREEANNOTATOR (Drummond et al., 2012) was used to estimate the final Maximum Clade Credibility Tree with a burn‐in of 10%. Trees were visualized and edited with FIGTREE 1.4.3. (Rambaut, 2016).

In order to more accurately estimate the time to the most recent ancestor (TMRCA), the clade with Cape Verdean haplotypes obtained from the previous analysis was re‐analyzed in BEAST.

Because evolutionary relationships among populations within species can be reticulate rather than bifurcating, haplotypes were connected on median‐joining networks followed by maximum parsimony to eliminate unnecessary median vectors and links (Bandelt, Forster, & Röhl, 1999) with the software NETWORK 5 available at website fluxus‐engineering.com. This program was also used to connect ND4 haplotypes from Cape Verde and Africa (54 sequences). In this case, we have applied a star contraction preprocedure.

### 2.3 Microsatellites genotyping and data analysis

The following 14 microsatellite loci were amplified by PCR using fluorescent‐labeled primers: A1, AC2, AC4, AC7, AG1, AG2, AG4, AG5, B2, B3, CT2 (Brown et al., 2011; Slotman et al., 2007), 12ACG1, 88AAT1, 201AAT1 (Lovin et al., 2009), and the corresponding amplification conditions. Amplified fragments were separated by capillary electrophoresis and sizes scored using the software GENE‐MARKER (SoftGenetics).

Allele richness (Rs) and private alleles richness (P) were calculated in HP‐RARE (Steven T. Kalinowski, 2004). Estimates of expected heterozygosity (He) and Hardy–Weinberg equilibrium (HWE) tests were performed with the software ARLEQUIN v.3.5 (Excoffier & Lischer, 2010). Linkage disequilibrium (LD) between pairs of loci was tested using the log‐likelihood ratio statistic available in GENEPOP v.4.2 (Raymond & Rousset, 1995; Rousset, 2008). To detect the presence of null alleles, we used the software MICRO‐CHECKER (Van Oosterhout, Hutchinson, Wills, & Shipley, 2004).

As larval sampling methods can lead to the collection of closely related individuals, we have used the maximum‐likelihood method in ML‐RELATE (Kalinowski, Wagner, & Taper, 2006) to calculate proportions of related individuals within each year, in order to confirm that the assumption of independent genotypes was not violated. For each pair of individuals, log‐likelihood was calculated for four relatedness categories: unrelated, parent–offspring, full‐siblings, and half‐siblings.

Estimates of current effective population size (Ne) were calculated by the bias‐corrected LD method (Waples & Do, 2010), using NeEstimator v.2.01 (Do et al., 2014). Because rare alleles may bias LD Ne estimates, alleles with frequency below 0.05 were not considered.

In order to detect recent population changes, Wilcoxon signed‐ranks tests of heterozygosity were performed with BOTTLENECK v.1.2.02 (Piry, Luikart, & Cornuet, 1999). Calculations were done under the stepwise mutation model (SMM) and a two‐phase model (TPM) with 30% of indels larger than one repeat, based on 1,000 replications.

Genetic differentiation between the two years was measured by pairwise Rst and Fst with ARLEQUIN v.3.5 (Excoffier & Lischer, 2010). Significance of estimates was assessed by 5,000 permutation tests.

Bayesian clustering analysis implemented in STRUCTURE v.2.3.4 (Pritchard, Stephens, & Donnelly, 2000) was performed using an admixture model, without prior information on sampling locations, assuming correlated allele frequencies among populations (*λ* was set at 1, default value). Twenty independent runs with 100,000 burn‐in steps and 500,000 iterations were done for each number of clusters (*K*). For this analysis, our data were combined with a global microsatellite data set from available in VectorBase/Project ID:VBS0061862 (Gloria‐Soria et al., 2016), using nine common loci (AC2, AC4, CT2, AG1, AG2, AG5, A1, B2, B3). K was tested from *K* = 1–5 at the global scale (81 populations in total) and from *K* = 1–10 at the African scale (23 African populations and subsequent analyses). The most likely value of *K* was determined with STRUCTURE HARVESTER (Earl & vonHoldt, 2012; Evanno, Regnaut, & Goudet, 2005). Results were visualized using CLUMPAK (Kopelman, Mayzel, Jakobsson, Rosenberg, & Mayrose, 2015).

In order to estimate levels of contemporary gene flow between Cape Verde and other African populations, we have also used the above‐mentioned microsatellite database to detect recent migrants with assignment tests performed with GENECLASS v.2 (Piry et al., 2004). The Bayesian‐likelihood criterion (Rannala & Mountain, 1997) and the as assignment criterion likelihood ratio (L): L_home/ L_max (Paetkau, Slade, Burden, & Estoup, 2004) was used with 1,000 simulated individuals.

Bonferroni corrections were used to adjust critical probability values for multiple tests (Rice, 1989).

### 2.4 *kdr *mutations

We have genotyped three *kdr* loci of the voltage‐gated sodium channel gene in the *A. aegypti* samples from Santiago Island, in search of the following five mutations: I1011M, I1011V, V1016I, V1016G, and F1534C. For the first four, we amplified and sequenced the intron spanning exons 20 and 21 (Saavedra‐Rodriguez et al., 2007). For F1534C, we performed a tetra primer PCR (Harris, Rajatileka, & Ranson, 2010).

## 3 RESULTS

### 3.1 ND4 mitochondrial gen*e*

The alignment of 360 bp of the ND4 gene sequenced in 42 Cape Verdean *A. aegypti* samples resulted in seven distinct haplotypes (Table S1). Some of these haplotypes matched sequences already published in GenBank: ND4‐1 (JQ926710‐Ivory Coast, KC800689‐Nigeria, KM042185‐Colombia); ND4‐2 (JN089748‐Brazil, JN896665‐Colombia); ND4‐3 (JN896664‐Colombia); ND4‐5 (AY906847‐Brazil, EF562501‐Cameroon, JQ926708‐Bolivia, JQ926709‐Ivory Coast, JQ926714‐Mexico, JQ926717‐Guinea). ND4‐4, ND4‐6, and ND4‐7 are unique haplotypes. This alignment includes 16 variable sites, two of which are parsimony informative, with a nucleotide diversity of *π* = 0.002 and a haplotype diversity of *h* = 0.609. Most of the substitutions were transitions (15), and there was a single transversion. Both neutrality tests for Santiago 2007 showed negative results but only Fu's FS was significant (Table 2). Mismatch analysis of Santiago 2007 gave a unimodal distribution of pairwise differences among haplotypes indicative of sudden expansion. The estimator τ (tau) was 0.762 (95% CI: 0.313–1.432), and the correspondent time since expansion (*T*) was 5,642 years (95% CI: 2,315–10,605), assuming a substitution rate of 0.0125 (Yu et al., 1999).

**Table 2:** Mitochondrial DNA genetic diversity and neutrality tests results of four populations of *Aedes aegypti* from Cape Verde

Gene	Analyses	Statistics	2007			2010	Mean	Global
			Santiago	Brava	Fogo	Santiago		
ND4	Genetic diversity	Hap	4	2	1	3	3	6
		Hd	0.471	0.600	NA	0.703	0.592	0.609
		Pi	0.001	0.002	NA	0.003	0.002	0.002
								*SD*
	Neutrality tests	Sample size	21	6	1	14	10.5	8.813
		Tajima's *D*	−1.299	1.445	0	1.459	0.401	1.324
		*D* *p*‐value	NS	NS	NA	NS	NS	NS
		Fu's FS	−2.387	0.795	0	0.783	0.202	1.503
		FS *p*‐value	0.005	NS	*N*.A	NS	NA	NA
COI								Global
	Genetic diversity	Hap	15	3	1	7	6.5	20
		Hd	0.949	0.733	NA	0.832	0.838	0.944
		Pi	0.004	0.001	NA	0.003	0.003	0.003
	Neutrality tests							*SD*
		Sample size	23	6	1	20	12.5	10.661
		Tajima's *D*	−1.195	−0.050	0	0.413	−0.208	0.690
		*D p*‐value	NS	NS	NS	NS	NS	NS
		Fu's FS	−9.777	−0.427	0	−0.496	−2.675	4.740
		FS *p*‐value	0.000	NS	NA	NS	NA	NA

Note

Number of haplotypes (Hap); haplotype diversity (Hd); nucleotide diversity (Pi); standard neutrality tests: Tajima's *D* (Tajima, 2009), Fu's Fs (Fu, 1997); standard deviation (*SD*) calculated for the haplotypes identified in Cape Verde; *p* values ≥ 0.05 were considered nonsignificant (NS); not applicable (NA).

The median‐joining network based on the ND4 gene revealed closely related haplotypes in a star‐like topology (Figure S1a). The central haplotype ND4‐1 was the most abundant (57%, Table S1) and the only present in the three islands sampled, while the remaining haplotypes had frequencies of less than 20%. Haplotype ND4‐2 was only found in Brava Island. Comparing the temporal samples of Santiago island, in 2010 we found two haplotypes previously detected in 2007 (ND4‐1 and ND4‐5) and one new haplotype (ND4‐7).

The alignment of these seven haplotypes with other published GenBank sequences for the same gene resulted in 368 sequences (Appendix S1a). This alignment included two sequences of *A. albopictus* (outgroup) and *A. aegypti* sequences from 26 countries, resulting in 96 haplotypes (Appendix S1b). The best fit model selected by both Akaike and Bayesian information criteria was GTR (Tavaré, 1986) with a proportion of invariable sites (*I* = 0.516) and a gamma shape parameter (*G* = 0.635) with 4 categories.

**Figure 2:**
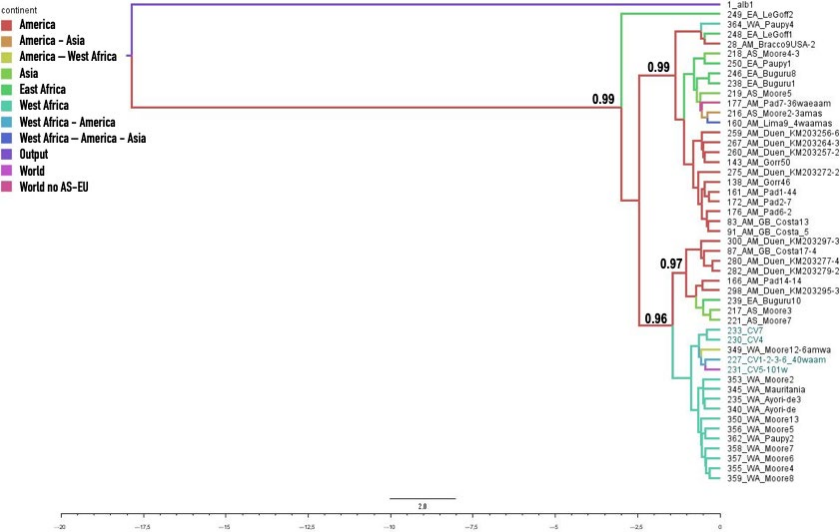
Bayesian phylogenetic tree (see text for details) of relationships among 49 haplotypes of *Aedes aegypti* based on a 360‐bp ND4 fragment and using *A. albopictus* as outgroup (in the Figure, it is written “output” by mistake). The support values written on the branches correspond to posterior probabilities (values < 0.900 are not presented). The haplotype codes and their origins are listed in Appendix S1c. The branch lines are colored by geographic distribution of the corresponding haplotypes. Time scale: substitutions/Myr

Given the low support of the clades in the initial analysis, possibly due to the high similarity of the, sequences, a new analysis was performed with the initial alignment of the 96 haplotypes (Appendix S1b) from which 47 South American sequences were eliminated (due to their overrepresentation in GenBank, Appendix S1c). The ND4 phylogenetic tree (Figure 2) showed two main clades supported by strong posterior probability values (i.e., >0.95). In the first one, haplotypes from Cape Verde occurred mainly associated with West African sequences, and some others from Asia and America, whereas the other clade contained most of East African sequences and other Asian and American sequences.

When the Cape Verdean and West African common clades (18 sequences, 16 haplotypes, Appendix S1d) were run alone in BEAST, we have obtained a height median (95% HPD) of 0.443 (0.257–0.686) corresponding to a TMRCA of 443,000 years (257,000–686,000).

From the initial set of 366 sequences (outgroups not considered), when the analysis was restricted to sequences of African origin (Figure S2), we detected a star‐like network with most haplotypes from West Africa in the center and the Cape Verdean haplotypes derived from the previous ones. In this network, many of East African haplotypes appear separated from most of West African and Cape Verdean ones by a higher number of mutations, and further apart from the center of the star.

### 3.2 COI mitochondrial gene

A fragment of 764 bp from the COI gene was sequenced in 50 *A. aegypti* individuals. The alignment resulted in 21 polymorphic sites defining 20 distinct haplotypes (Table S2). Only the haplotype COI‐1 matched one sequence already published in GenBank: JQ926693‐Ivory Coast, and the other 19 haplotypes seem to be unique. All observed substitutions were transitions. This alignment includes 20 variable sites, nine of which are parsimony informative, with *π* = 0.003 and *h* = 0.943. Both neutrality tests for Santiago 2007 showed negative results but only Fu's FS was significant (Table 2). The mismatch analysis in Santiago 2007 revealed a unimodal distribution of pairwise differences among haplotypes consistent with a model of sudden expansion. τ‐tau was 3.031 (95% CI: 2.041–4.133) and the correspondent time since expansion *T* = 5,741 years (95% CI: 3,866–7,827), assuming a substitution rate of 0.023 (Brower, 1994).

The median‐joining network based on the COI gene revealed also related haplotypes in a star‐like topology (Figure S1b). Haplotype COI‐15 in the center of the star was the only present in the three sampled islands. All except one haplotype (COI‐7) were present in Santiago Island. COI‐7 was only detected in Brava. In 2010, we have found four new haplotypes undetected in 2007 (COI‐17, 18, 19, 20), while other three haplotypes were also present in the past sample of 2007 (COI‐4, 6, 13).

The alignment of the 20 haplotypes with other published 74 GenBank sequences from 24 countries for the same gene, resulted in 94 sequences of 493 bp (Appendix S2). This alignment resulted in 67 haplotypes with a nucleotide diversity of *π* = 0.01671, 98 variable sites, 37 of which are parsimony informative, 61 singleton variable sites. The best fit model selected by the Bayesian information criteria was TN (Tamura & Nei, 1993) with a proportion of invariable sites (*I* = 0.7948) and gamma shape parameter (*G* = 1.073).

**Figure 3:**
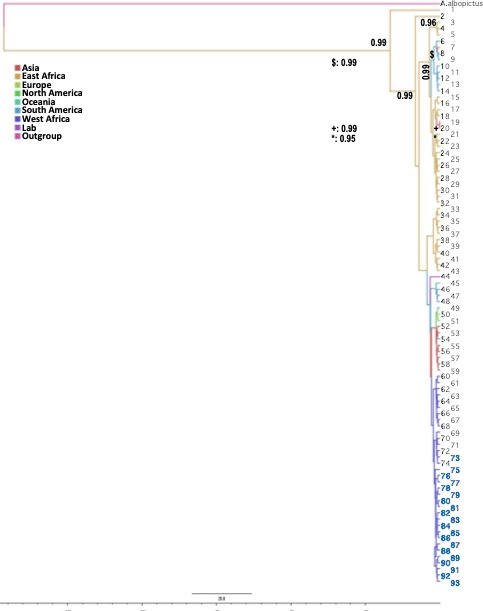
Bayesian phylogenetic tree (see text for details) of relationships among 94 sequences of *Aedes aegypti* based on a 493‐bp COI fragment and using *A. albopictus* as outgroup. The support values written on the branches correspond to posterior probabilities (values < 0.800 are not presented). The haplotype codes and their origins are listed in Appendix S2. The branch lines are colored by geographic distribution of the corresponding haplotypes. Time scale: substitutions/Myr. *A. albopictus*: 155albopictus_JQ004524; 1: 100Mdgr_HQ688298; 2: 190TZ_KX446465; 3: 186TZ_KX446459; 4: 169U4_KX446426; 5: 183TZ_KX446456; 6: 027Mex_JQ926698; 7: 078Thail_JQ926692; 8: 008BR_JQ926703; 9: 025Mart_JQ926697; 10: 026Mart_JQ926697; 11: 003Bol_JQ926678; 12: 002Bol_JQ926677; 13: 023Guyana_HQ688297; 14: 001Bol_JQ926676; 15: 102TZA_JQ926704; 16: 189TZ_KX446464; 17: 187TZ_KX446460; 18: 180U10_KX446447; 19: 086Vietn_JQ926686; 20: 087Vietn_JQ926687; 21: 172U5_KX446430; 22: 192TZ_KX446469; 23: 089Eur_HQ688296; 24: 173U5_KX446431; 25: 191TZ_KX446467; 26: 175U9_KX446436; 27: 185TZ_KX446458; 28: 168U4_KX446425; 29: 181U11_KX446450; 30: 184TZ_KX446457; 31: 188TZ_KX446463; 32: 182U12_KX446451; 33: 178U10_KX446442; 34: 171U4_KX446429; 35: 165U2_KX446420; 36: 166U4_KX446423; 37: 174U9_KX446434; 38: 179U10_KX446444; 39: 177U9_KX446438; 40: 170U4_KX446427; 41: 176U9_KX446437; 42: 164U1_KX446418; 43: 167U4_KX446424; 44: 154Formosus_AY056597; 45: 011Col_KM203198; 46: 012Col_KM203248; 47: 028Mex_JQ926699; 48: 010Col_KM203140; 49: 052FP_HQ688295; 50: 111Madeira_KF909122; 51: 036USA_JQ926684; 52: 085Vietn_JQ926685; 53: 077Thail_JQ926691; 54: 040Ven_JQ926701; 55: 048Camb_JQ926690; 56: 084Vietn_HQ688292; 57: 046Camb_JQ926688; 58: 047Camb_JQ926689; 59: 045Camb_HQ688294; 60: 145RCI_JQ926694; 61: 163Ben1_KX446415; 62: 118Cam_JQ926702; 63: 144RCI_JQ926693; 64: 161Ben1_KX446401; 65: 142Guin_JQ926700; 66: 157Ben2_KX446393; 67: 141Guin_HQ688293; 68: 160Ben1_KX446397; 69: 162Ben1_KX446413; 70: 156Ben2_KX446392; 71: 159Ben1_KX446396; 72: 146RCI_JQ926695; 73: 121CV_COI03; 74: 158Ben1_KX446395; 75: 135CV_COI17; 76: 130CV_COI12; 77: 120CV_COI02; 78: 124CV_COI06; 79: 134CV_COI16; 80: 136CV_COI18; 81: 122CV_COI04; 82: 129CV_COI11; 83: 123CV_COI05; 84: 132CV_COI14; 85: 131CV_COI13; 86: 138CV_COI20; 87: 119CV_COI01; 88: 125CV_COI07; 89: 128CV_COI10; 90: 127CV_COI09; 91: 133CV_COI15; 92: 126CV_COI08; 93: 137CV_COI19

The resulting COI Bayesian tree (Figure 3) showed two main clades. Haplotypes from Cape Verde were all concentrated in one of the clades grouping with all other West African sequences and some Central African ones. The other clade included all the Eastern Africa lineages. American and Asian sequences were present in both clades.

When the Cape Verdean and West African common clades (25 sequences, Appendix S2b) were run alone in BEAST, we have obtained a height median (95% HPD) of 0.126 (0.061–0.238) corresponding to a TMRCA of 126,000 years (61,000–238,000).

### 3.3 Microsatellite genetic diversity

We genotyped 70 mosquitoes from Santiago Island, 47 individuals collected in 2007 and 23 in 2010. The 14 loci were polymorphic with allele richness ranging from 4 to 8 and private allele richness from 0 to 4. The expected heterozygosity varied between 0.494 and 0.783 (Table S3). Regarding HWE tests (Table S3), we have found three significant tests in 2007 (A1, AG4, and 12ACG1) and two in 2010 (AG1 and 201AAT1), all due to heterozygote deficit. None of the loci deviated from HWE expectations in both samples. Null alleles were suspected in four loci (A1, 12ACG1, 88AAT1, and 201AAT1). The microsatellites used in this study have been extensively validated in previous studies (Brown et al., 2011; Gloria‐Soria et al., 2016; Lovin et al., 2009) where deviations from HWE were associated with null alleles, but at frequencies that did not affect the assessment of population structure. Eleven out of 182 (6%) pairwise associations of loci showed significant LD but no pair of loci was consistently linked in both samples. The percentage of unrelated individuals was 87% in 2007 and 82% in 2010 (i.e., no alleles among pairs of individuals were identical by descent, Table S4).

The two temporal samples showed low but significant (*p* < 0.0001) genetic differentiation with Rst = 0.027 and Fst = 0.043. Estimates of current effective population size decreased from 55 in 2007 to 8 in 2010, with nonoverlapping 95% confidence intervals, indicative of significant differences in Ne between years (Table 3). Tests performed with BOTTLENECK v.1.2.02 (Piry et al., 1999) revealed significant heterozygote excess in the sample of Cape Verde 2010 under the TPM (Table 3).

**Table 3:** Estimates of effective population size and heterozygosity tests for *Aedes aegypti* from Cape Verde

Populations	Ne	CI	Bottleneck	
			P(SMM)	P(TPM)
CV2007	54.8	39.0–84.5	NS	NS
CV2010	7.7	5.5–10.6	NS	0.0017

Note

Ne: current effective population size based on the bias‐corrected LD method (Waples & Do, 2013); CI: parametric 95% confidence interval; P(SMM) and P(TPM): *p‐*value for heterozygote excess under SMM and TPM, after Bonferroni correction; NS: nonsignificant.

### 3.4 Population structure

**Figure 4:**
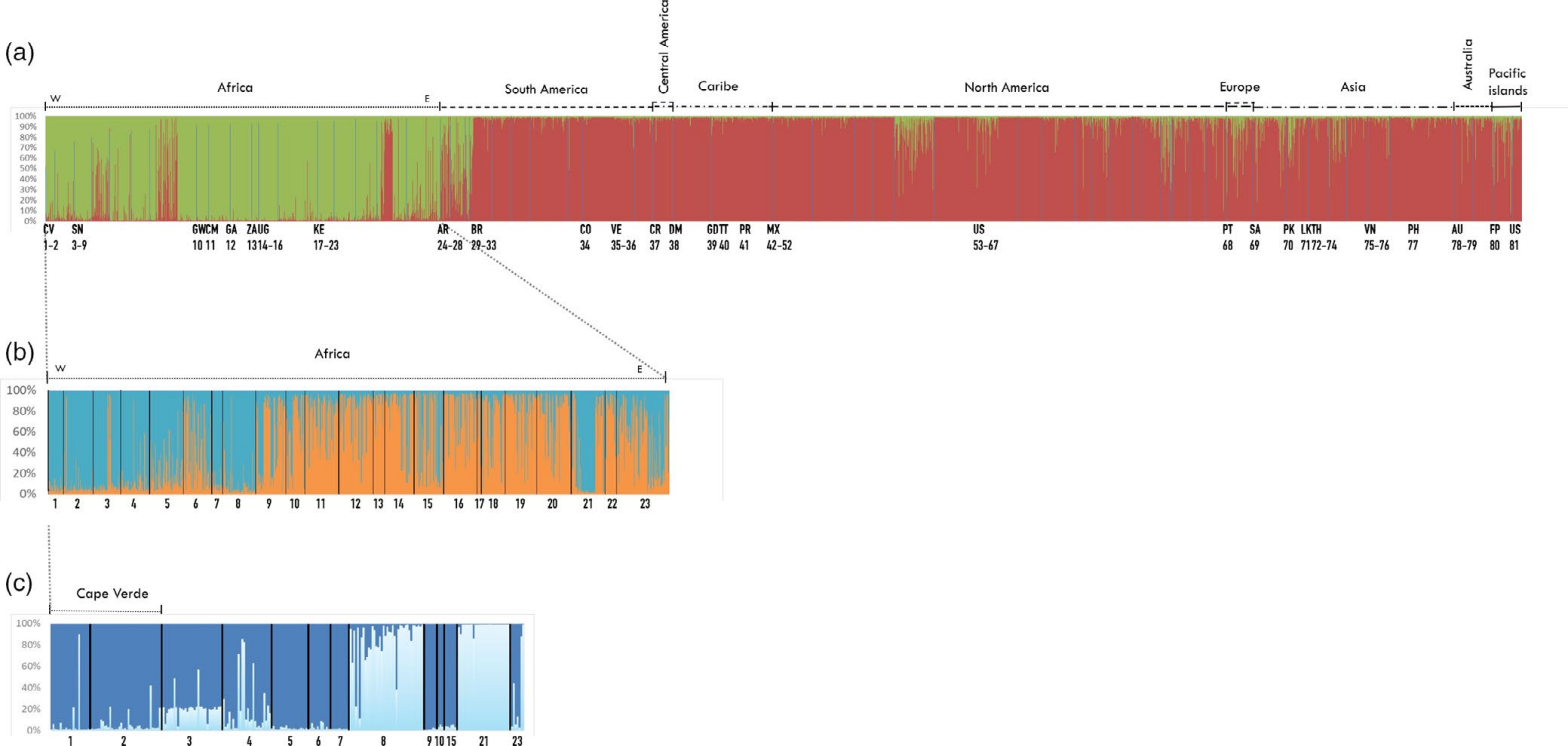
Global genetic structure of *Aedes aegypti*. STRUCTURE bar plots indicating genetic groupings of different geographic locations based on nine microsatellite loci. Each vertical bar represents an individual. The height of each bar represents the probability of assignment (*Q*) to each of *K* = *N* clusters as determined using the Delta K method. Each cluster is indicated by different colors. (a) Genetic groupings of 81 geographic locations and 3,703 individuals, sorted by countries, best *K* = 2 clusters, *A. a. aegypti*: red and *A. a. formosus*: green. Population code numbers are as follows: 1‐Santiago 2010, CV; 2‐Santiago 2007, CV; 3‐Dakar, SN; 4‐N'goye, SN; 5‐Sedhiou, SN; 6‐Koungheul, SN; 7‐Ngari, SN; 8‐Goudiry, SN; 9‐PK‐10, SN; 10‐Bijagos, GW; 11‐Yaounde, CM; 12‐Francesville, GA; 13‐Johannesburg, ZA; 14‐Kichwamba, UG; 15‐Bundibugyo, UG; 16‐Lunyo, UG; 17‐Kakamenga, KE; 18‐Kisimu, KE; 19‐Nairobi, KE; 20‐Rabai‐out, KE; 21‐Rabai‐in, KE; 22‐Garissa, KE; 23‐Mombasa, KE; 24‐Cordoba, AR; 25‐Salta, AR; 26‐Iguazu, AR; 27‐La Plata, AR; 28‐Posadas, AR; 29‐Cachoeiro, BR; 30‐Maraba, BR; 31‐Natal, BR; 32‐Jacobina, BR; 33‐Rio de Janeiro, BR; 34‐Cali, CO; 35‐Bolivar, VE; 36‐Zulia, VE; 37‐Siquirres, CR; 38‐Dominica, DM; 39‐Carriacou, GD; 40‐Trinidad, TT; 41‐Patillas, PR; 42‐Tijuana, MX; 43‐Pijijiapan, MX; 44‐Las Palomas, MX; 45‐Lomas de Zapatero, MX; 46‐Iguala, MX; 47‐Mazatan, MX; 48‐Tapachula Norte, MX; 49‐Hermosillo, MX; 50‐Nogales, MX; 51‐Chetumal, MX; 52‐Amacuzac, MX; 53‐Maricopa, USA; 54‐Madera, USA; 55‐Clovis, USA; 56‐New Orleans, USA; 57‐Houston, USA; 58‐Vaca Key, USA; 59‐Miami, USA; 60‐Musco, USA; 61‐San Mateo, USA; 62‐Rio FL, USA; 63‐Exeter, USA; 64‐Los Angeles, USA; 65‐Conch Key, USA; 66‐Palm Beach, USA; 67‐North Key West, USA; 68‐Madeira, PT; 69‐Riyadh, SA; 70‐Pakistan, PK; 71‐Sri Lanka, LK; 72‐Rayong, TH; 73‐Prachuabkhirikan, TH; 74‐Bangkok, TH; 75‐Ho Chi Minh, VN; 76‐Hanoi, VN; 77‐Cebu, PH; 78‐Townsville, AU; 79‐Cairns, AU; 80‐Tahiti, FP; 81‐Hawaii, USA. All details about the non‐Cape Verdean samples are described elsewhere (Gloria‐Soria et al., 2016). (b) Genetic groupings of 23 African populations and 989 individuals from a), sorted by countries and by longitude (W: west to E: east), best *K* = 2 clusters (blue and orange). (c) The same 989 individuals from 23 African populations as in b), sorted by *Q*. (d) Genetic groupings of 13 African populations and 258 individuals selected from individuals with the *Q* > 0.90 of assignment to the blue cluster in a), best *K* = 2

Figure 4 illustrates the results of the clustering analysis implemented by STRUCTURE v.2.3.4 (Pritchard et al., 2000). The best *K* for the global data set with 81 world populations (Figure 4a) was two clusters corresponding to the two subspecies *A. aegypti aegypti* and *A. aegypti formosus*, as seen previously (Gloria‐Soria et al., 2016). All individuals from Cape Verde belonged to the *formosus* cluster. When only the samples from Africa were analyzed (Figure 4b), two clusters were obtained, roughly separating most of the samples from Cape Verde, Senegal, and a few from Kenya (blue cluster: populations 1–9 from West Africa, 21 and 23 from East Africa), from the rest (orange cluster). In order to better detail this clustering result, we selected individuals from the blue cluster with a probability of assignment (Q)> 0.90 re‐analyzed them in STRUCTURE v.2.3.4 (Pritchard et al., 2000) with the same previous conditions. The result is presented in Figure 4c, including 59 out of the original 71 Cape Verdean samples. The best *K* was again *K* = 2, but this time, the Senegalese population of Goudiry (8 in Figure 4) grouped with the Kenyan Rabai‐outdoor collection (21 in Figure 4), leaving the rest to another cluster, where Cape Verde was incorporated.

GENECLASS v.2 (Piry et al., 2004) assigned only one potential contemporary migrant from Goudiry, Senegal (population 8 in Figure 4) to Cape Verde 2010, with a *L* = 4.090. No migrants from Cape Verde to continental Africa were detected.

### 3.5 kdr mutations

The analysis of the *kdr* gene from 74 individuals revealed that the *A. aegypti* population from Cape Verde was monomorphic with 100% of wild‐type alleles at the three loci analyzed (i.e., 1011I, 1016V, and 1534F).

## 4 DISCUSSION

Phylogeographic and population genetic analysis of *A. aegypti* from Cape Verde Islands revealed the main elements of the invasion history and recent demographic events of this insular mosquito population. Our results suggest an ancient West African origin and no evidence of recent founder events. We report comparable levels of genetic diversity, within the range of values for other African populations for both mitochondrial (Bennett et al., 2016; Paupy et al., 2008) and nuclear markers (Gloria‐Soria et al., 2016). However, there was a low but significant intertemporal genetic differentiation associated with a signal of population bottleneck and a reduction in effective population size in 2010. The target site mechanism of knockdown resistance was likely not present in the population of Santiago island in 2007 and 2010.

### 4.1 West African origin

Clustering analysis of microsatellite data indicates that the Cape Verdean *A. aegypti* population belongs to the subspecies *formosus*. This result agrees with previous analyses based on morphological data (Vazeille et al., 2013). Microsatellite data also suggest a West African origin, possibly having *A. aegypti* from Senegal as the source population. The levels of expected heterozygosity are within the average of African populations (Gloria‐Soria et al., 2016) and similar to the only African insular sample from Guinea‐Bissau (sample 10‐Bijagos, GW in Figure 4). All other island populations analyzed in that study showed lower levels of genetic diversity than Cape Verde.

The matrilineages of *A. aegypti* from Cape Verde occurred in a basal clade mainly associated with West African mosquito populations for both ND4 and COI (Figures 2 and 3). Some of the most abundant haplotypes had matching sequences with West African samples from Ivory Coast, Nigeria, Cameroon, and Guinea. The levels of nucleotide diversity (*π*) are similar to values reported in West Africa for both COI and ND4 (Bennett et al., 2016; Paupy et al., 2008), and much lower than the values reported for East Africa (Bennett et al., 2016), America (Eskildsen et al., 2018; Jaimes‐Dueñez, Arboleda, Triana‐Chávez, & Gómez‐Palacio, 2015; Scarpassa, Cardoza, & Cardoso, 2008; Twerdochlib et al., 2012), and Asia (Bosio et al., 2005), possibly due to multiple introduction events in those continents (Bracco, Capurro, Lourenço‐de‐Oliveira, & Sallum, 2007; Powell & Tabachnick, 2013).

### 4.2 Invasion history and expansion time

Our analysis suggests that the TMRCA of Cape Verdean and West African mtDNA lineages is in the late Pleistocene (ND4Median = 443,000 years, COI Median = 126,000 years, 95% HPD = 61,000–686,000).

In the three Cape Verdean islands surveyed, we found several unique haplotypes very closely related in a star‐like network in both ND4 and COI (Figure S1). Given the highest haplotype diversity in Santiago Island and ubiquity of central haplotypes in the trees (ND4‐1 and COI‐15 in Figure S1), it is likely that the colonization of the more western and smaller islands of Fogo and Brava was made from the island of Santiago (Figure 1). However, a more detailed history of these founder events is not possible due to the reduced sampling. It would be interesting to collect mosquito samples from the other islands to unveil the complete history of *A. aegypti* in the archipelago. Cape Verde has a geological age of 25.6 Myr and is spread over 58,000 km^2^ in two diverging chains. With older islands in the east and younger ones in the west, like Santiago Island dating to 10 mya (Duarte & Romeiras, 2009), possible different colonization scenarios can be expected.

Focusing on Santiago Island from 2007, besides the already mentioned star‐like tree of very close haplotypes, we found a concordant unimodal mismatch distribution of mutations and also a significantly negative Fu's FS neutrality test (Table 2). Together, these are genetic signatures of population expansion (Fu, 1997; Harpending, 1994). The expansion time calculated for both genes overlapped, suggesting that expansion may have started at around 5,700 years ago. This estimate predates the occupation of the island of Santiago by humans, in the 15th‐century AD. Therefore, genetic evidence suggests that *A. aegypti* invaded Santiago Island in the early Holocene without the aid of humans. This would correspond to a colonization of the ancestral sylvatic and zoophilic *A. aegypti formosus*, possibly blood feeding on available reptile (Heatwole & Shine, 1976) or bird (Tandon & Ray, 2000) hosts already present on the island. In Cape Verde, there is evidence for reptile presence before 6.2 mya in the late Miocene (Carranza, Arnold, Mateo, & López‐Jurado, 2001) and bird species establishment during the Pleistocene (2.6–0.01 mya) (Bourne, 1951).

Cape Verde played an important role in the historical slave trade network (Lobban, 2018). Between the 16th and 17th century, Santiago island was an obligatory stop for ships going from the coast of Angola, São Tomé, Ghana, and Guinea‐Bissau to Brazil and the West Indies (Lobban, 2018; Russell‐Wood, 1998). This could have promoted ancient gene flow with continental Africa and the introduction of *A. aegypti* from Cape Verde in the New World by passive transportation. However, both mtDNA and microsatellites point to the presence of an *A. a. formosus* population in Cape Verde, which does not agree with the hypothesis of an early African domestication of *A. aegypti aegypti* followed by subsequent expansion to the Americas. Furthermore, Cape Verdean mtDNA haplotypes for both ND4 and COI cluster in subclades composed almost exclusively by West African haplotypes, suggesting that recent gene flow between Cape Verde and the Americas has been infrequent. Nevertheless, knowing that the first step for the out‐of‐Africa dispersion of *A. aegypti* was from the African West coast (Crawford et al., 2017; Kotsakiozi et al., 2018) toward the Americas during the Atlantic slave trade, one cannot rule out the possibility that the long‐established population of *A. aegypti* in Cape Verde could have contributed to the gene pool of the founding populations in the New World. It would be important to compare Cape Verde with additional populations from Senegal and Angola to better understand the relative contribution of each of these *A. a. formosus* populations to the founding of *A. a. aegypti* in the Americas.

### 4.3 Evidence of recent demographic perturbation

When comparing samples from 2007 and 2010 from Santiago Island, there was significant temporal genetic differentiation associated with a signal of population bottleneck and a significant reduction in effective population size in the sample of 2010. Although the reduced sample size of 2010 could have influenced estimates, these observations could also be a consequence of the increased vector control efforts implemented during the late 2009 dengue outbreak. In response to the epidemic, local health authorities have intensified treatment of breeding sites with temephos and indoor/outdoor spraying with deltamethrin (Dia et al., 2012; Rocha et al., 2015). This could have affected the effective population size of the local *A. aegypti* population. Absence of *kdr* mutations in both the 2007 and 2010 samples is in line with a field study carried out in November 2009 that revealed susceptibility of *A. aegypti* from Cape Verde to pyrethroid insecticides (Dia et al., 2012). However, this situation has changed in 2012 and 2014, with the first reports of resistance to these insecticides (Rocha et al., 2015). This reinforces the hypothesis of insecticide‐based reduction of Ne of the mosquito population, and the increase of selection pressure for insecticide resistance during the 2009 dengue epidemic.

### 4.4 Epidemic preparedness and implications for vector control

The role of airports and airlines in the spread of vector‐borne diseases has been helpful in predicting the risks of vector‐borne disease importation and establishment (Huang, Das, Qiu, & Tatem, 2012; Tatem et al., 2012). Cape Verde is in a strategic route linking Africa and South America. The major Dengue outbreak in Cape Verde was caused by a DENV3 virus that originated from Senegal (Franco et al., 2010) and the local Zika outbreak had its most likely source in Brazil (WHO, 2016). In 2015, Cape Verde received more than 7,000 travelers from Zika virus‐affected countries, including direct flights from Brazil (Bogoch et al., 2016; Lourenço et al., 2018). Therefore, the risk of introduction of new virus species/strains and new vectors (e.g., *A. albopictus*) is real and surveillance should thus be prioritized. The challenges imposed by the recent Dengue and Zika epidemics highlight the need for a well‐trained healthcare workforce capacitated for rapid response under emergency situations caused by vector‐borne diseases.

Although mutations associated with *kdr* resistance have not yet been reported in Cape Verde, the first records of insecticide resistance recently detected in Santiago island raise caution for the sustainability of insecticide‐based vector control. Integrated vector management is being implemented in Cape Verde given that the country is in the pre‐elimination stage for malaria (Ministry of Health Cape Verde et al., 2012) and has recently suffered from Dengue and Zika outbreaks (WHO, 2009, 2015). In this context, the adoption of alternative noninsecticidal vector control strategies should be considered a priority for the Cape Verde health authorities.

## CONFLICT OF INTEREST

None declared.

## Data Availability

Data for this study are available at GenBank (MK359818‐MK359844) and VectorBase (VBP0000346) (Salgueiro et al., 2019).
