# Supplementary material for: Phylogeography and invasion history of Aedes aegypti, the Dengue and Zika mosquito vector in Cape Verde islands (West Africa)
Source: Evol Appl. 2019 Aug 3;12(9):1797–811. doi: 10.1111/eva.12834 (PMC6752157; doi:10.1111/eva.12834)
Supplement: Supplementary file 3 [file EVA-12-1797-s003.docx]

# Manuscript id: EVA-2018-194-OA.R2

# Manuscript title: Phylogeography and invasion history of Aedes aegypti, the Dengue and Zika mosquito vector in Cape Verde islands (West Africa)

# Supporting Material

## Table S1

| **Table S1.** Variable nucleotide positions within the 360-bp sequence of ND4 analyzed in 42 *Aedes aegypti* mosquitoes from Cape Verde. | | | | | | | | | | | | | | | | | | | | | | | | | | |
| --- | --- | --- | --- | --- | --- | --- | --- | --- | --- | --- | --- | --- | --- | --- | --- | --- | --- | --- | --- | --- | --- | --- | --- | --- | --- | --- |
|  |  |  |  | **16 Positions of variable nucleotides in base pairs (bp)** | | | | | | | | | | | | | | | |  | **2007** | | | |  | **2010** |
| **Haplotypes** | **N** | **%** |  | **6**  **3**  **6** | **6**  **4**  **2** | **6**  **4**  **8** | **6**  **8**  **4** | **6**  **9**  **3** | **7**  **5**  **3** | **7**  **6**  **2** | **7**  **7**  **1** | **8**  **1**  **0** | **8**  **5**  **5** | **8**  **8**  **8** | **9**  **2**  **4** | **9**  **2**  **7** | **9**  **3**  **3** | **9**  **4**  **2** | **9**  **7**  **6** |  | **S** | **B** | **F** | **T** |  | **S** |
| Reference  sequence |  |  |  | **C** | **C** | **G** | **A** | **C** | **T** | **C** | **G** | **C** | **C** | **G** | **T** | **G** | **T** | **G** | **A** |  |  |  |  |  |  |  |
| ND4-1 | 24 | 57 |  | T | T | . | . | T | C | T | A | T | T | A | . | . | . | . | C |  | 14 | 3 | 1 | 18 |  | 6 |
| ND4-2 | 3 | 7 |  | T | T | A | . | T | C | T | A | T | T | A | . | . | . | . | C |  | 0 | 3 | 0 | 3 |  | 0 |
| ND4-3 | 1 | 2 |  | T | T | . | G | T | C | T | A | T | T | A | . | . | . | . | C |  | 1 | 0 | 0 | 1 |  | 0 |
| ND4-4 | 1 | 2 |  | T | T | . | G | T | C | T | A | T | T | A | . | A | . | . | C |  | 1 | 0 | 0 | 1 |  | 0 |
| ND4-5 | 8 | 19 |  | T | T | . | . | T | C | T | A | T | T | A | . | . | C | . | C |  | 4 | 0 | 0 | 4 |  | 4 |
| ND4-6 | 1 | 2 |  | T | T | . | . | T | C | T | A | T | T | A | . | . | . | A | C |  | 1 | 0 | 0 | 1 |  | 0 |
| ND4-7 | 4 | 10 |  | T | T | . | . | T | C | T | A | T | T | A | C | . | C | . | C |  | 0 | 0 | 0 | 0 |  | 4 |
| Total | 42 |  |  |  |  |  |  |  |  |  |  |  |  |  |  |  |  |  |  |  |  |  |  | 28 |  | 14 |
| Position number is indicated above each base and haplotype name, number of individuals sequenced (N) and the frequency (%) on the left of each sequence.  Dots indicate identity with corresponding base of the first sequence. Only polymorphic positions are shown, and these are numbered with reference to the published sequence of ND4 from *Aedes aegypti* clone AET-3506 ND4 DQ440274.  The second part of the table indicates the distribution and frequency of these haplotypes in the sampled islands: S-Santiago, F- Fogo, B-Brava, T-Total.  Some of the above haplotypes matched sequences already published in GenBank: ND4-1 (JQ926710-Ivory Coast, KC800689-Nigeria, KM042185-Colombia); ND4-2 (JN089748-Brazil, JN896665-Colombia); ND4-3 (JN896664-Colombia); ND4-5 (AY906847-Brazil, EF562501-Cameroon, JQ926708-Bolivia, JQ926709-Ivory Coast, JQ926714-Mexico, JQ926717-Guinea), ND4-4,6 and 7 are unique haplotypes. | | | | | | | | | | | | | | | | | | | | | | | | | | |

## Table S2

| **Table S2** Variable nucleotide positions within the 764-bp sequence of COI analyzed in 50 *Aedes aegypti* mosquitoes from Cape Verde. | | | | | | | | | | | | | | | | | | | | | | | | | | | | | | | |
| --- | --- | --- | --- | --- | --- | --- | --- | --- | --- | --- | --- | --- | --- | --- | --- | --- | --- | --- | --- | --- | --- | --- | --- | --- | --- | --- | --- | --- | --- | --- | --- |
|  |  |  |  | **21 Positions of variable nucleotides in base pairs (bp)** | | | | | | | | | | | | | | | | | | | | | **2007** | | | | |  | **2010** |
| **Haplotypes** | **N** | **%** |  | **2**  **5**  **5** | **2**  **7**  **3** | **3**  **0**  **0** | **3**  **2**  **1** | **3**  **5**  **7** | **3**  **6**  **9** | **4**  **5**  **3** | **5**  **0**  **7** | **5**  **1**  **3** | **5**  **1**  **6** | **5**  **4**  **0** | **6**  **0**  **6** | **6**  **3**  **0** | **6**  **6**  **3** | **6**  **6**  **9** | **6**  **7**  **5** | **6**  **9**  **9** | **7**  **1**  **4** | **7**  **4**  **1** | **7**  **8**  **9** | **8**  **9**  **7** |  | **S** | **B** | **F** | **T** |  | **S** |
| Reference |  |  |  | **A** | **C** | **T** | **G** | **G** | **G** | **T** | **A** | **G** | **A** | **C** | **G** | **C** | **C** | **A** | **C** | **C** | **C** | **C** | **G** | **T** |  |  |  |  |  |  |  |
| COI-1 | 3 | 6 |  | . | T | . | . | . | . | . | . | . | . | . | A | . | . | . | T | . | . | . | . | . |  | 3 |  |  | 3 |  |  |
| COI-2 | 1 | 2 |  | . | . | . | A | . | . | . | . | . | . | . | A | . | . | . | . | . | . | . | . | . |  | 1 |  |  | 1 |  |  |
| COI-3 | 1 | 2 |  | . | . | . | . | A | A | . | . | . | . | . | A | . | . | . | T | . | . | . | . | . |  | 1 |  |  | 1 |  |  |
| COI-4 | 4 | 8 |  | . | . | . | . | A | . | . | G | . | . | . | A | . | . | . | T | . | . | . | . | . |  | 2 |  |  | 2 |  | 2 |
| COI-5 | 1 | 2 |  | . | . | . | . | . | A | . | . | . | . | . | A | . | . | . | T | . | . | . | . | . |  | 1 |  |  | 1 |  |  |
| COI-6 | 5 | 10 |  | . | . | . | . | . | . | . | G | . | . | T | A | . | . | . | T | . | . | . | . | . |  | 4 |  |  | 4 |  | 1 |
| COI-7 | 1 | 2 |  | . | . | . | . | . | . | . | . | A | . | . | A | . | . | . | T | . | . | . | . | . |  |  | 1 |  | 1 |  |  |
| COI-8 | 1 | 2 |  | . | . | . | . | . | . | . | . | . | G | . | A | T | . | . | T | T | . | . | . | . |  | 1 |  |  | 1 |  |  |
| COI-9 | 1 | 2 |  | . | . | . | . | . | . | . | . | . | . | . | A | T | T | . | T | . | . | . | . | . |  | 1 |  |  | 1 |  |  |
| COI-10 | 1 | 2 |  | . | . | . | . | . | . | . | . | . | . | . | A | . | T | . | T | . | . | T | . | . |  | 1 |  |  | 1 |  |  |
| COI-11 | 3 | 6 |  | . | . | . | . | . | . | . | . | . | . | . | A | . | . | G | T | . | . | . | . | . |  | 1 | 2 |  | 3 |  |  |
| COI-12 | 1 | 2 |  | . | . | . | . | . | . | . | . | . | . | . | A | . | . | . | . | . | . | . | . | . |  | 1 |  |  | 1 |  |  |
| COI-13 | 4 | 8 |  | . | . | . | . | . | . | . | . | . | . | . | A | . | . | . | T | . | T | . | . | . |  | 1 |  |  | 1 |  | 3 |
| COI-14 | 3 | 6 |  | . | . | . | . | . | . | . | . | . | . | . | A | . | . | . | T | . | . | T | . | . |  | 3 |  |  | 3 |  |  |
| COI-15 | 5 | 10 |  | . | . | . | . | . | . | . | . | . | . | . | A | . | . | . | T | . | . | . | . | . |  | 1 | 3 | 1 | 5 |  |  |
| COI-16 | 1 | 2 |  | . | . | . | . | . | . | C | G | . | . | . | A | . | . | . | T | . | . | . | . | . |  | 1 |  |  | 1 |  |  |
| COI-17 | 4 | 8 |  | G | . | C | . | . | . | . | . | . | . | . | A | . | . | . | T | . | . | . | . | . |  |  |  |  |  |  | 4 |
| COI-18 | 1 | 2 |  | . | . | . | . | A | . | . | G | . | . | T | A | . | . | . | T | . | . | . | . | . |  |  |  |  |  |  | 1 |
| COI-19 | 2 | 4 |  | . | . | . | . | . | . | . | . | . | . | . | A | . | . | . | T | . | T | . | A | . |  |  |  |  |  |  | 2 |
| COI-20 | 7 | 14 |  | . | . | . | . | . | . | . | . | . | . | . | A | . | . | . | T | . | . | . | . | C |  |  |  |  |  |  | 7 |
|  | 50 | 100 |  |  |  |  |  |  |  |  |  |  |  |  |  |  |  |  |  |  |  |  |  |  |  | 23 | 6 | 1 | 30 |  | 20 |
| Position number is indicated above each base and haplotype name, number of individuals sequenced (N) and the frequency (%) on the left of each sequence. Dots indicate identity with corresponding base of the first sequence. Only polymorphic positions are shown, and these are numbered with reference to the published sequence of COI from *Aedes aegypti* GenBank accession No. AY056597 (strain Formosus). The second part of the table indicates the distribution and frequency of these haplotypes in the sampled islands: S-Santiago, F- Fogo, B-Brava, T-Total.  Only the haplotype COI-1 matched one sequence already published in GenBank: JQ926693-Ivory Coast | | | | | | | | | | | | | | | | | | | | | | | | | | | | | | | |

## Table S3

**Table S3** Summary statistics for 14 microsatellite loci in *Aedes aegypti* from Cape Verde

| **Sampling sites** |  | **A1** | **AG1** | **AG5** | **B2** | **AC7** | **CT2** | **AC2** | **AG2** | **AC4** | **AG4** | **12ACG1** | **88AAT1** | **201AAT1** | **B3** | **All loci** | |
| --- | --- | --- | --- | --- | --- | --- | --- | --- | --- | --- | --- | --- | --- | --- | --- | --- | --- |
|  |  |  |  |  |  |  |  |  |  |  |  |  |  |  |  | **mean** | **total** |
| Santiago 2007 (N=47) | Rs | 4 | 5 | 5 | 5 | 7 | 5 | 5 | 7 | 4 | 5 | 5 | 6 | 4 | 5 | 5 | 72 |
|  | P | 0 | 0 | 0 | 1 | 0 | 1 | 2 | 4 | 0 | 1 | 0 | 3 | 2 | 2 | 1 | 16 |
|  | He | **0.643** | 0.796 | 0.660 | 0.771 | 0.696 | 0.736 | 0.472 | 0.753 | 0.633 | **0.724** | **0.526** | 0.809 | 0.713 | 0.689 | 0.687 |  |
| Santiago 2010 (N=23) | Rs | 5 | 5 | 5 | 5 | 8 | 4 | 4 | 5 | 4 | 5 | 5 | 4 | 3 | 3 | 5 | 64 |
|  | P | 1 | 0 | 0 | 1 | 0 | 0 | 0 | 0 | 0 | 0 | 0 | 0 | 0 | 0 |  | 2 |
|  | He | 0.662 | **0.769** | 0.724 | 0.709 | 0.805 | 0.696 | 0.587 | 0.663 | 0.703 | 0.672 | 0.644 | 0.733 | **0.275** | 0.565 | 0.658 |  |
| All samples (N=70) | Rs | 4 | 5 | 5 | 5 | 8 | 4 | 5 | 6 | 4 | 5 | 5 | 6 | 4 | 5 | 5 | 72 |
|  | He | 0.652 | 0.783 | 0.692 | 0.740 | 0.751 | 0.716 | 0.530 | 0.708 | 0.668 | 0.698 | 0.585 | 0.771 | 0.494 | 0.627 | 0.673 |  |
| *N*: sample size; Rs: Allelic richness on minimum sample size of 18 diploid individuals; P: Private allele richness; He: Expected heterozygosity; In bold: significant *P-*value for HWE test after Bonferroni correction. | | | | | | | | | | | | | | | | | |

## Table S4

| **Table S4** Percentage of individuals within each *Aedes aegypti* temporal sample  from Cape Verde assigned to the four relatedness categories | | | | |
| --- | --- | --- | --- | --- |
| **Population** | **U** | **HS** | **FS** | **PO** |
| 2007 | 87% | 11% | 2% | 0% |
| 2010 | 82% | 9% | 5% | 3% |
| U=unrelated, FS=full-siblings, HS=half-siblings, and PO=parent-offspring | | | | |

## Appendix 1_ND4_excel file

## Fig. S1

## Fig. S1(A)


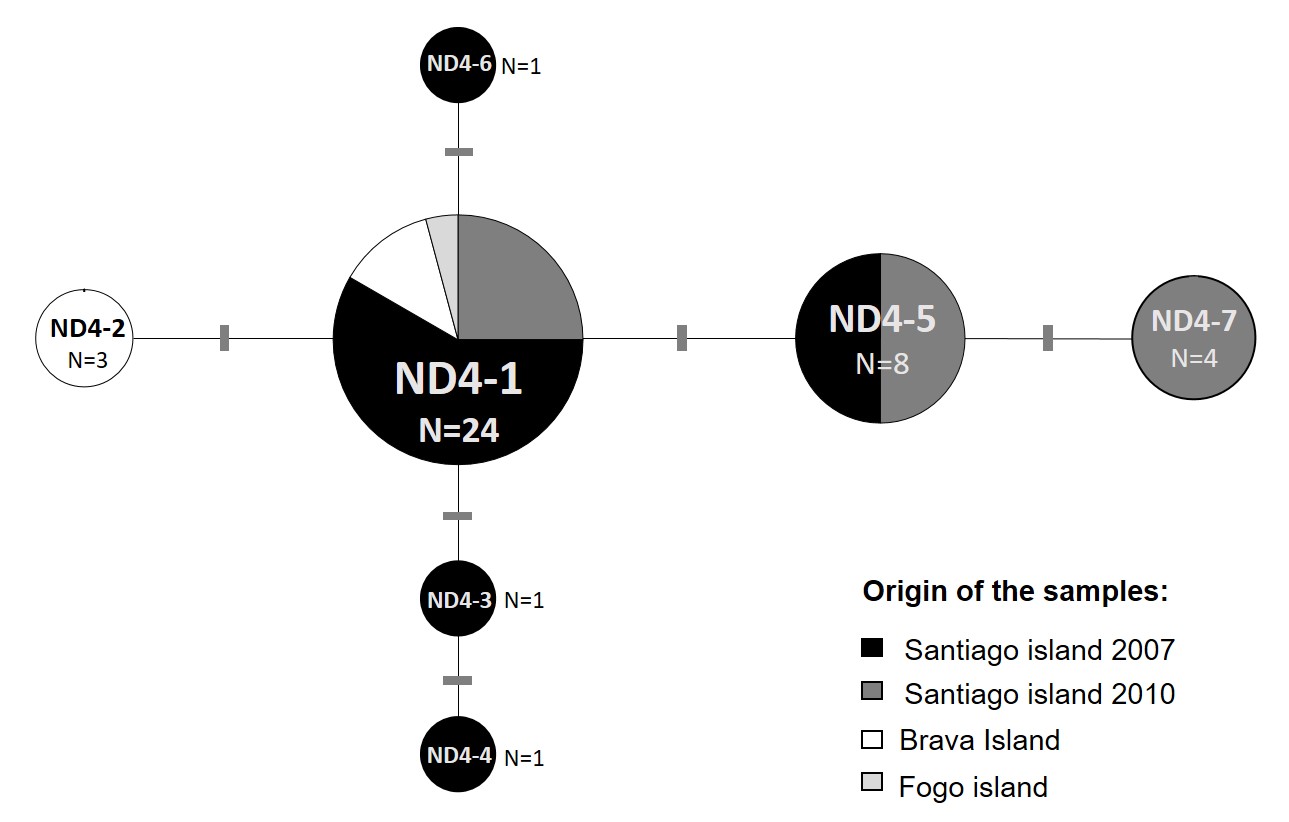


## Fig. S1(B)


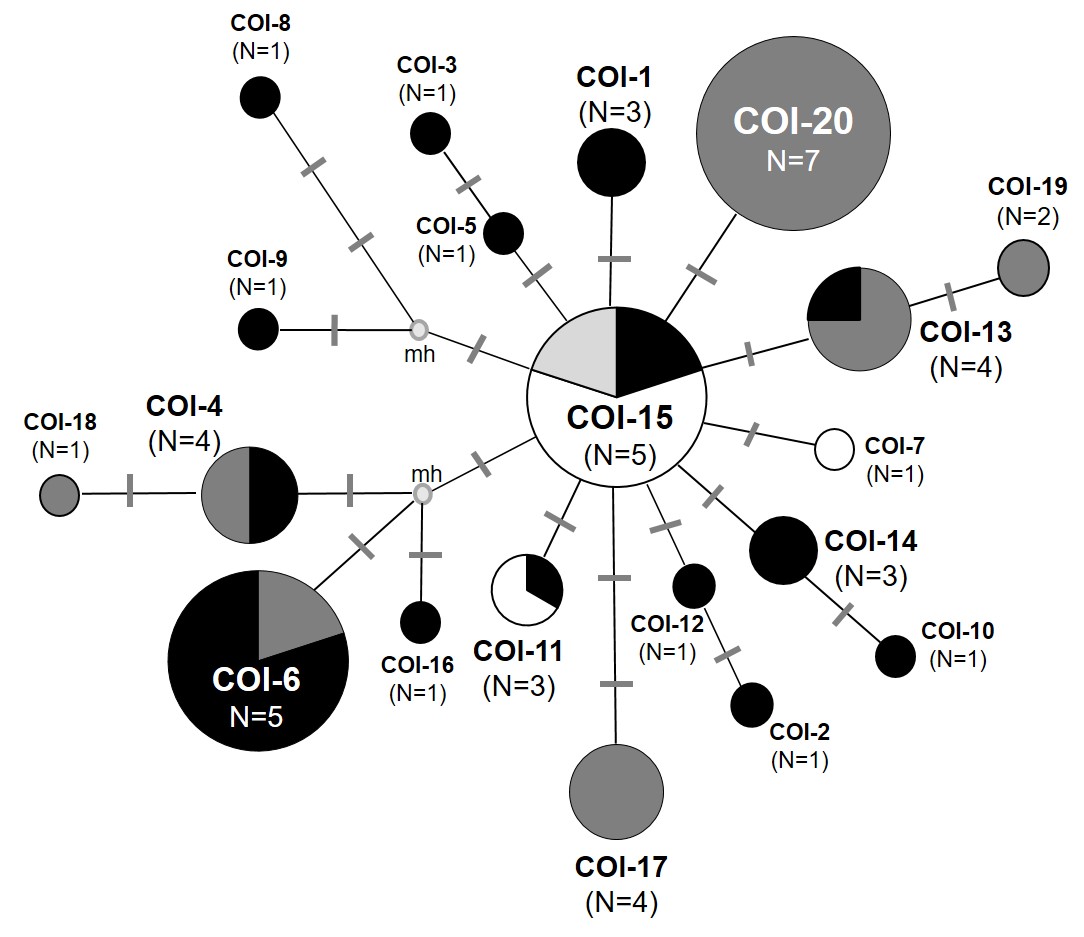


## Legend: Fig. S1

Median–joining networks of *A. aegypti* from Cape Verde based on: (A) seven haplotypes from 360-bp of the ND4 gene sequenced over 42 individuals; (B) 20 haplotypes from 764-bp of the COI gene sequenced over 50 individuals. The area of circles is proportional to the frequency of haplotypes. Each grey dash in between the lines connecting the haplotypes represents one mutation. The frequency and geographic distribution of each haplotype is also presented, *mh* is the abbreviation for missing haplotype.

## Fig. S2


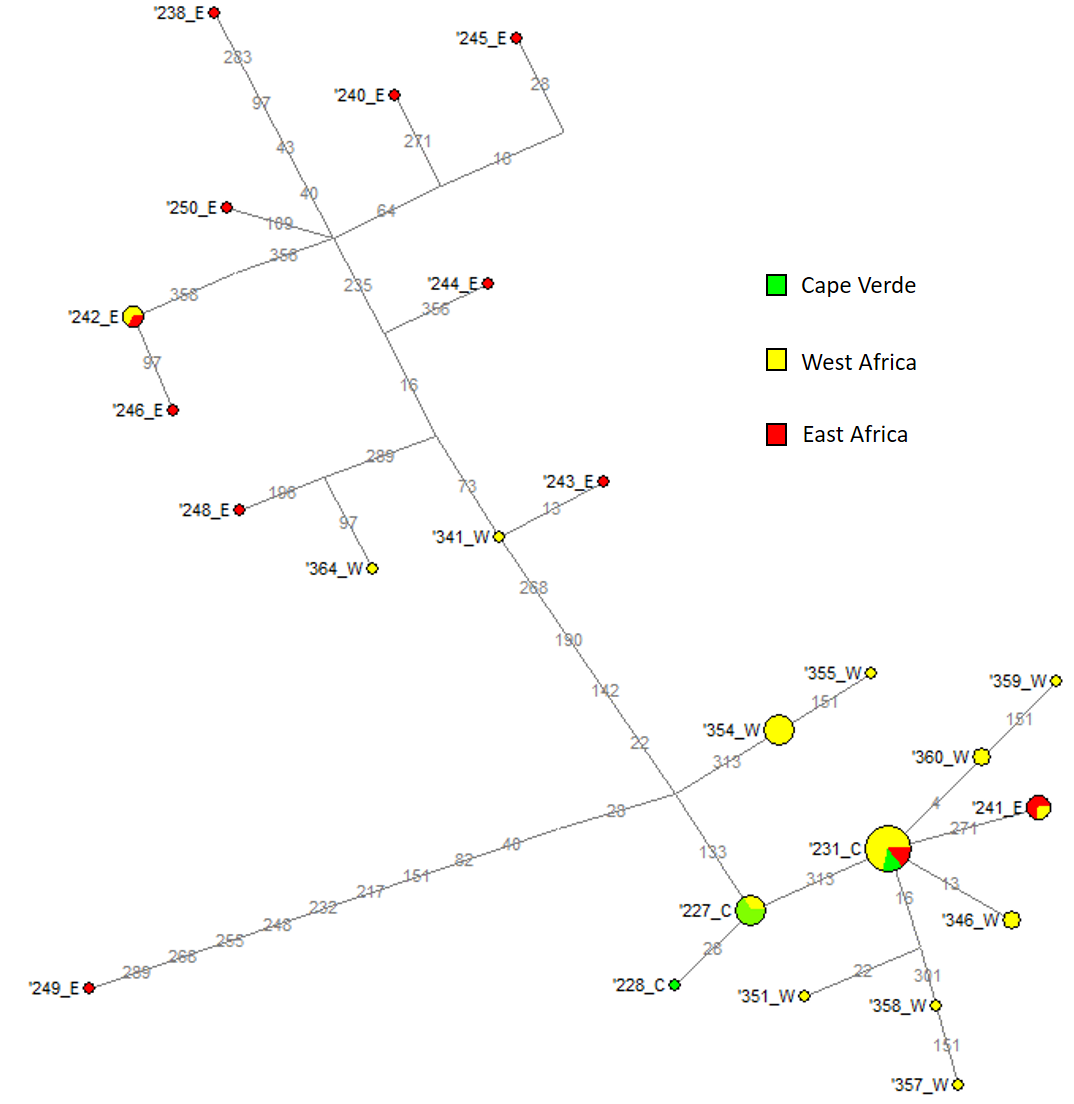


## Legend: Fig. S2

Median–joining network based on 54 sequences from 360-bp of the ND4 gene sequenced of *A. aegypti* from Cape Verde (C), East Africa (E) and West Africa (W). Each grey number between the lines connecting the haplotypes represents one mutation. The geographic distribution of each haplotype is represented by colours: green-C, yellow-W, red-E.

The area of each circle is proportional to the haplotype frequency. The major circles include more than one sequence: 231_C also includes 13 other sequences 233, 236, 237, 340, 342, 343, 348, 353, 356, 361, 362, 365, 368; 227_C also includes six other sequences 229, 230, 232, 234, 366; 354_W also includes six other sequences 235, 349, 350, 363, 367; 242_E also includes sequences 24, 214. All other circles represent one single sequence. The information and references on the sequences and the country of origin are listed on Appendix 1.

## Appendix 3_COI_excel file
